# Neonatal Sepsis, Antibiotic Susceptibility Pattern, and Treatment Outcomes among Neonates Treated in Two Tertiary Care Hospitals of Yangon, Myanmar from 2017 to 2019

**DOI:** 10.3390/tropicalmed6020062

**Published:** 2021-04-28

**Authors:** Nan Aye Thida Oo, Jeffrey K. Edwards, Prajjwal Pyakurel, Pruthu Thekkur, Thae Maung Maung, Nant San San Aye, Hla Myat Nwe

**Affiliations:** 1 Department of Medical Research, Ministry of Health and Sports, Yangon 11191, Myanmar; themgmg.dr@gmail.com; 2 Department of Global Health, University of Washington, Seattle, WA 98195, USA; jeffrey.edwards@fmridaho.org; 3 School of Public Health and Community Medicine, B.P. Koirala Institute of Health Sciences, Dharan 56700, Nepal; prajjwal.pyakurel@bpkihs.edu; 4 Centre for Operational Research, International Union against Tuberculosis and Lung Disease (The Union), 68 Boulevard Saint Michel, 75006 Paris, France; Pruthu.TK@theunion.org; 5 Neonatal Intensive Care Unit, Central Women Hospital, Yangon 11121, Myanmar; nssaye@gmail.com; 6 Department of Neonatology, Yangon Children Hospital, Yangon 11191, Myanmar; drhmnwe@gmail.com

**Keywords:** antibiotic resistance, late onset sepsis, Gram-negative sepsis, operational research, neonatal intensive care unit (NICU)

## Abstract

Neonatal sepsis is a leading cause of morbidity and mortality in developing countries. This study aimed to assess the proportion of culture-confirmed sepsis, bacteriological pathogen profile, culture report turnaround times, antibiotic susceptibility patterns, and treatment outcomes of all with neonatal sepsis admitted in two tertiary care hospitals in Yangon, Myanmar, 2017–2019. This was a cross sectional study utilizing a standardized electronic database and paper-based records. Bacteriological profiles and associated factors were analyzed with descriptive statistics and Poisson Regression. Of those with suspected sepsis, 42% were bacteriologically confirmed and 74% of confirmed sepsis was resistant to at least first-line antibiotics. Neonates with late onset sepsis (LOS) (aPR: 1.2 (95% CI: 1.1–1.4, *p* = 0.008)) were more likely to have bacteriologically confirmed sepsis (45%) versus early onset sepsis (38%). Gram-negative organisms were most commonly isolated (63%), associated with multidrug-resistant organisms and with a high case-fatality rate (64%). These findings suggest that enhanced national guidance regarding infection control and prevention, antibiotic stewardship, and first-line antibiotic choices need to be provided. The link between LOS with infection and prevention protocols needs to be further explored in this context to decrease sepsis risk, neonatal mortality, and reduce further antimicrobial resistance.

## 1. Introduction

Neonatal sepsis is a leading cause of morbidity and mortality in the pediatric age group and, despite recent advances for neonatal care, there has been limited progress in mortality reduction [1]. Neonatal sepsis is the eighth leading cause of under-five mortality globally, accounting for more than one million deaths globally [2]. The World Health Organization (WHO) estimates that neonatal sepsis has an incidence of 7–38/1000 live births in Asia [2]. It remains one of the three most frequent causes of neonatal death in developing countries [3] and is a focus of the United Nations’ Sustainable Development Goal #3 [4].

In Myanmar, 25% of all under five deaths are neonatal and 68% of neonatal deaths occur during the first week of life. Neonatal sepsis accounts for 26% of all neonatal deaths [5]. There has been a decrease in the neonatal mortality rate (NMR) within Myanmar (from 41 in 1998 to 25 per 1000 live births in 2016). However, the Myanmar NMR remains one of the highest among Asian countries [5].

Neonatal sepsis can be categorized as early onset sepsis (EOS) and late onset sepsis (LOS) [6]. The source of infection for EOS is highly associated with the organisms that are carried in the maternal genital tract leading to vertical transmission, while LOS is typically caused by either nosocomial (hospital-acquired) or community-acquired sources [6]. 

Newborns frequently present with no specific signs or symptoms for neonatal sepsis, the diagnosis and treatment can be especially challenging for developing countries [7]. Blood culturing with drug sensitivity testing is the only current standard diagnostic tool for neonatal sepsis in resource constrained settings. However, this approach is often time-consuming and can result in delaying the appropriate treatment. In addition, there are factors influencing culture results, such as the volume of blood required, low levels of bacteremia, and laboratory technical limitations in isolating pathogens [8]. In developed countries, the ratio of culture-positive and culture-negative sepsis ranges from 1:6 to 1:16 [9] and culture-positivity rates range from 6.7% to 55.4%, globally [8].

The profile of organisms causing neonatal septicemia vary in different geographical areas and within hospitals of the same region [10]. The causative pathogens for neonatal sepsis are frequently different when comparing developing versus developed countries. Bacterial pathogens such as *Klebsiella pneumoniae*, *Staphylococcus aureus*, and coagulase-negative *Staphylococcus* (CoNS) are often the leading causes of neonatal sepsis in developing countries [10]. These organisms are associated with resistance to commonly used antibiotics, which can lead to challenges in choosing first-line antimicrobials [11,12]. Additionally, common pathogens such as Gram-positive bacteria, as a cause of neonatal sepsis, may be replaced by others over time, particularly in developing countries [12].

There are no large studies that we are aware of determining the burden and factors (maternal and neonatal) associated with neonatal sepsis, and also describing the isolated pathogens and antibiotic susceptibility patterns in tertiary care hospitals of Myanmar. The studies from the past on prevalence of neonatal sepsis and its associated factors were limited to single hospital settings with small sample sizes [13,14,15,16]. Only one study has reported on isolated pathogens and antibiotic susceptibility patterns from early onset neonatal sepsis in Yankin Children’s Hospital’s (YCH) neonatal intensive care unit (NICU) in Myanmar [17]. 

Knowledge regarding common pathogens and antimicrobial susceptibility patterns causing neonatal septicemia is critical in order to select the most appropriate antibiotic therapy to decrease neonatal morbidity and mortality. This is especially important in tertiary facilities, where antimicrobial resistance (AMR) is likely to be higher. This study aimed to assess the proportion of neonatal sepsis, bacteriological pathogen profile, culture report turnaround times, antibiotic susceptibility patterns, and treatment outcomes among those with neonatal sepsis admitted in two tertiary care hospitals in Myanmar from January 2017 to December 2019. 

## 2. Materials and Methods

### 2.1. Study Design

A cross sectional study utilizing routinely collected clinical data from neonatal cases admitted to two tertiary care hospitals.

### 2.2. Setting

#### 2.2.1. General Setting

Myanmar is a country located in South East Asia. It is bordered by the Bay of Bengal, Andaman Sea, Gulf of Thailand, and the countries of Bangladesh, India, China, Laos, and Thailand. Healthcare services in Myanmar are mainly funded by the government sector. From 1962 to 2011, the former government spent 0.5% to 3% of the country’s GDP on healthcare. In 2017, the reformed government spent 5.2% of GDP on healthcare expenditures [18]. Myanmar is divided into seven states and regions with a population of approximately 54 million. 

#### 2.2.2. Health Care Setting 

The Department of Health is responsible for providing comprehensive health care services to the entire country. Curative services are provided by various public and private health institutions. There are General hospitals, Specialist hospitals, Teaching hospitals, Region/State hospitals, District hospitals, and Township hospitals in urban areas. To increase the average life expectancy in Myanmar, the Ministry of Health has focused increasingly upon reducing the child and infant mortality rates.

#### 2.2.3. Health Care Facility and Neonatal Care in Myanmar

Public hospitals in Myanmar are categorized into general hospitals (up to 2000 beds), specialist hospitals and teaching hospitals (100–1200 beds), regional/state hospitals and district hospitals (200–500 beds), and township hospitals (25–100 beds). In rural areas, sub-township hospitals and station hospitals (16–25 beds), rural health centers (no beds), and sub-rural health centers (no beds) provide healthcare, including public health services. There are 1056 public hospitals with 56,748 beds in total [19]. 

The Newborn and Child Survival Forum has been held regularly, with the purpose of sharing information on newborn and child survival related issues, including prioritization of interventions, establishment of a database for all child survival research studies, and identification of gaps [20]. All tertiary and secondary hospitals have paediatricians for neonatal care and followed the guidelines distributed by Myanmar Paediatric Society. However, there is no paediatrician at the town care level. Standard treatment protocols on managing common newborn conditions have been developed with support from tertiary care hospital paediatric providers. 

#### 2.2.4. Specific Setting

##### Yangon Children Hospital

Yangon Children’s Hospital (YCH) is in Yangon City in Myanmar. The estimated population of the city was 7.3 million in 2019, and 23.4% are children 0–14 years of age. YCH is the largest tertiary paediatrics center in Southern Myanmar with 550 inpatient beds including a NICU. The hospital receives approximately 120,000 outpatient visits and 25,000 admissions each year. Care is provided to all children <15 years old from Yangon City and other provinces of Southern Myanmar. The neonatology department of this hospital care for neonates born outside of the hospital, which is called extramural birth. There are estimated 1200–1300 neonatal ward admissions and 200–300 clinically suspected neonatal sepsis cases per year (Hospital profile, 2018).

##### Central Women Hospital

Yangon Central Women’s Hospital (CWH) is the largest maternity facility in Myanmar with an annual delivery rate of 12000–15000. It is also a tertiary referral center and teaching hospital, affiliated with the University of Medicine (1). CWH has 800 beds including a NICU and there are approximately 2000–2500 babies admitted to the NICU per year.

#### 2.2.5. Laboratory Services

All collected blood cultures were sent to either the laboratory inside the hospital or a private laboratory. Both laboratory settings have the ability to perform antibiotic susceptibility testing by disk diffusion using guidelines established by the Clinical and Laboratory Standards Institute [21] and the VITEK 2 COMPACT automated system.

#### 2.2.6. Neonatal Sepsis Treatment

All the suspected cases of neonatal sepsis are treated with ampicillin and gentamicin, or amikacin and cefotaxime, as first-line drugs for empiric treatment regimens (Guidelines from Myanmar Paediatric Society). Sulperazone, meropenem, and vancomycin are considered as second-line or reserve groups. Antibiotic treatment was changed or modified according to antimicrobial resistance pattern per the culture and drug susceptibility testing (CDST) report.

### 2.3. Operational Definitions

Operational definitions are shown in Table 1.

### 2.4. Study Population and Period

In this study, all neonates with a diagnosis of clinically suspected neonatal sepsis and who were an inpatient at YCH or CWH during the period of June 2017 to December 2019 were included in the study. For neonates with several episodes of sepsis during prolonged hospital stays, the first episode of sepsis was included in the study.

### 2.5. Laboratory Procedures

All blood cultures were collected from a peripheral vein with proper aseptic precautions before starting any antibiotic therapy. A minimum of 1 mL of blood was collected from each neonate with proper aseptic precautions and inoculated immediately into 9 mL of brain heart infusion broth with 0.025% Sodium polyanethol sulfonate as anticoagulant (HiMedia Laboratories, Mumbai). Blood culture bottles were incubated aerobically at 37 °C for 7 days. Subcultures were made on sheep blood agar and MacConkey agar (HiMedia, Mumbai, India), and chocolate agar routinely after 48 h and 7 days. Subculture was also done in between, if visible turbidity appeared. All the inoculated culture plates were incubated at 37 °C overnight. Antibiotic susceptibility was performed according to the Clinical and Laboratory Standards Institute (CLSI) [21]. Minimal Inhibitory Concentration (MIC) tests were performed to determine the susceptibility to vancomycin. Vancomycin susceptibility was screened by using vancomycin MIC (≥8 ug/mL) in an automated system and vancomycin 6 mg/mL concentration for Agar dilution method. Antimicrobial resistance patterns were noted as susceptible, intermediate or resistant for each individual antibiotic.

### 2.6. Data Variables and Collection

Data were extracted from the clinical records of studied neonates within the neonatal units of CWH and YCH hospitals. Social demographic information of mother and neonate, and clinical data including: age in days on admission, gender, onset of neonatal sepsis, risk factors for sepsis, date of admission, date of birth, mother’s name, mode of delivery, gestational age at delivery, history of prolonged rupture of membranes (PROM), meconium-stained liquor, foul smelling liquor, and birth asphyxia were extracted from “SEARO neonatal-perinatal database” which has the details of all the neonates admitted to the hospitals since 2016. Blood culture submitted (Yes/No), date of blood culture taken, date of receiving the culture results, name of isolated organism, antimicrobial resistance to common drugs, multidrug-resistance (MDR) organisms, and treatment outcome were collected from “Neonatal Sepsis Register Book” with all details related to isolated organisms, culture, sensitivity, and treatment outcomes of all those with suspected neonatal sepsis.

### 2.7. Analysis and Statistics

Data extracted from the neonatal sepsis register were entered using EpiData entry software version 3.1 (EpiData Association, Odense, Denmark). The EpiData database and SEARO electronic database downloaded in Microsoft Excel format were merged using hospital number. Data was analyzed using Stata 14.0 (StataCorp LP, College Station, TX, USA).

The baseline demographic, perinatal, and clinical characteristics of the neonates with clinically suspected sepsis and bacteriological profile, AMR pattern among six most common Gram-positive and negative bacteria were summarized using frequency and percentage. The ratio of MDR organisms associated with case fatality rate among neonates with bacteriologically confirmed sepsis was also summarized using frequency and percentage.

A modified Poisson regression model with robust variance estimator was utilized to assess the association between demographic, perinatal, and clinical characteristics with confirmed sepsis among those who had CDST results. Similar models were used to assess demographic, perinatal, clinical characteristics associated with resistance to at least one first-line antibiotic, and unfavorable treatment outcomes (died, left against medical advice, requested discharge) among neonates with bacteriologically confirmed sepsis. The prevalence ratio (PR) and adjusted PR (aPR) were used as the measure of association.

### 2.8. Ethics Approval

The study proposal was approved by the Ethics Review Committee, Department of Medical Research (approval no Ethics/DMR/2019/129 of 20/10/2019) and the Union Ethical Advisor Group, Paris, France (approval no 62/19 of 13/08/2019). Written permission from each specific hospital was obtained through official administrative channels. This study only utilized hospital clinical records and the name of parents/caretakers of the study neonates was not included in data entry and analysis. All identifying data was anonymized.

## 3. Results

### 3.1. Characteristics of Study Participants

There were 10,935 NICU admissions, of which 1705 (15.6%) were neonates with suspected neonatal sepsis admitted from January 2017 to December 2019 to either YCH or CWH and included in this study (Table 2). Of the total neonates, most were ≤3 days old on admission (1235, 72.4%) and were males (1008, 59.1%). The majority of neonates were delivered spontaneously in a hospital (1484, 87.0%) and without surgical or mechanical intervention (861, 50.5%). In addition, these neonates were largely delivered by doctors (1433, 84.0%) rather than by other health professionals and did not have maternal history of prolonged rupture of membranes in most cases (1264, 74.1%).

Further analysis revealed that admission age in days, gestational age, place/mode of delivery, provider type, foul smelling liquor, and birth weight of neonates showed significant differences between the two hospitals. However, there were no significant differences for the gender of neonates, PROM, and early versus late onset of sepsis (Table 2).

### 3.2. Culture and Drug Susceptibility Testing (CDST) Uptake, Bacteriological Confirmation, Resistance to First-Line Antibiotics, and Outcomes among Neonates with Clinically Suspected Sepsis

**Figure 1:**
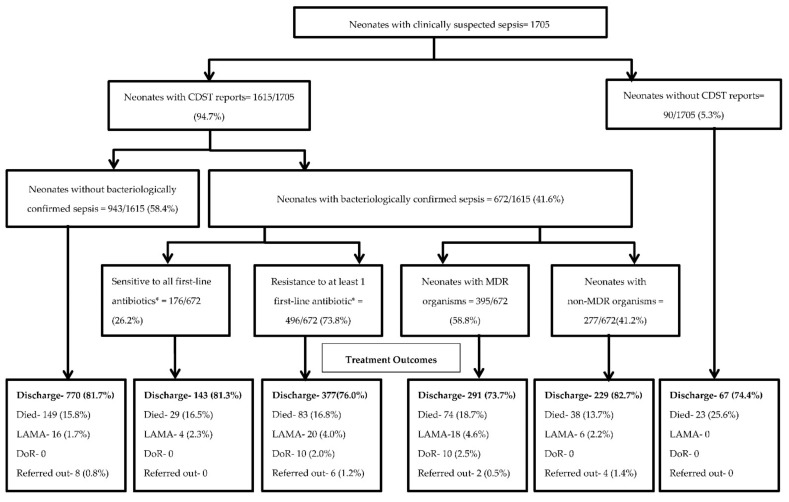
Flowchart on CDST uptake, bacteriological confirmation, resistance to first-line antibiotics, and outcomes among neonates with clinically suspected sepsis admitted to the NICU of two tertiary care hospitals in Yangon, Myanmar from January 2017 to December 2019. CDST = culture and drug susceptibility testing; NICU = neonatal intensive care unit; LAMA = Left against medical advice; DoR = discharge on request. * First line antibiotics-ampicillin, amikacin, gentamicin, and cefotaxime.

Of 1705 suspected cases of neonatal sepsis, 1615 (94.7%) had blood cultures reported (Figure 1). We found there were 672/1615 (41.6%) neonates with bacteriologically confirmed sepsis. Among neonates with confirmed sepsis, 496/672 (73.8%) were resistant to at least one first-line antibiotic (ampicillin, amikacin, gentamicin, or cefotaxime). The proportion of the deceased neonates without CDST reports was 23/90 (25.6%). The proportion of deaths between the neonates with antibiotic resistance compared to those neonates without resistance were similar, 16.8% vs 16.5%, respectively (Figure 1). 

### 3.3. Associated Risk Factors Related to Neonatal Septicaemia with Positive Culture

The association of risk factors of those neonates with bacteriologically confirmed sepsis is shown in Table S3. The proportion of neonates found to be septic was significantly higher in the year 2017 (aPR: 1.4 (95% CI: 1.2–1.7) and 2018 (aPR: 1.5 (95% CI: 1.3–1.8) compared to 2019 (*p* < 0.001). There was no difference in the proportion of those with bacteriologically confirmed sepsis when comparing home versus institutional place of delivery. Compared to normal delivery, those neonates delivered through emergency CS (aPR: 1.2 (95% CI: 1.1–1.4) were more likely to have bacteriologically confirmed sepsis. Lastly, those with late onset sepsis (aPR: 1.2 (95% CI: 1.1–1.4, *p* = 0.008) were more likely to have confirmed sepsis compared to early onset sepsis (Table S3). 

### 3.4. Turn-Around Time of CDST among Neonates with Clinically Suspected Sepsis

For neonates who had reported blood culture results (1533), the median number of days for blood cultures to be obtained following admission was two days (Table 3). The median days from culture collection to sensitivity reporting was six days and 89.3% of them were reported within 14 days. 

### 3.5. Magnitude and Bacterial Profiles and Antibiotic Susceptibility Pattern among Neonatal Septicaemia

Among neonates whom CDST was completed, 672 (41.6%) showed bacterial growth (Figure 1). For those with bacteriologically confirmed sepsis, Gram-negative species (421/672, 62.6%) were more common than Gram-positives species (251/672, 37.4%) (Table S4). 

The most commonly isolated pathogens were CoNS (both *Staphyloccus epidermidis* and CoNS others) (149/672, 22.2 %) and *Klebsiella pneumoniae* (90/672, 13.4%), followed by *Staphylococcus aureus* (73/672, 10.9 %) (Table S1). The predominant Gram-positive pathogens included *S. aureus*, *S. epidermidis*, and CoNS (others). *Streptococci* spp. were isolated in only a few neonates. 

The most common Gram-negative pathogens were *K. pneumoniae*, *Acinetobacter* spp., *Serratia marcescens*, and *Enterobacter* spp. (Table S1). All *Klebsiella* spp. combined accounted for 16.1% (108/672) of the total isolated pathogens. Of all the culture-positive pathogens, 43.7% (294) comprised early-onset neonatal sepsis and 56.3% (378) late-onset neonatal sepsis. Gram-positive pathogens were seen to be equally distributed in both EOS and LOS (46.6%, 117/251 and 53.4%, 134/251), whereas Gram-negative pathogens were predominant in LOS (58.0%, 244/421 versus 42.0%, 177/421) (Table S2*)*.

*S. epidermitis* was more common in LOS (70.6%) compared to EOS (29.4%) whereas *K. pneumoniae* was more common in LOS than EOS (61.1% vs 38.9%) (Table S2). Gram-positive isolates had high resistance rates to ampicillin (78.4%), amoxicillin-clavulanic acid (64.2%), and cefotaxime (42.9%). Of note, 48.1% of Gram-positive bacteria were resistant to piperacillin-tazobactam and 14.4% of them were resistant to vancomycin (Table 4).

Susceptibility patterns in Gram-negative isolates demonstrated a high resistance to amoxicillin-clavulanic acid (69.4%), ceftazidime (63.5%), gentamicin (62.4%), and amikacin (32.6%). Additionally, antibiotic resistance was particularly high for *K. pneumoniae* (the most common pathogen isolated) with significant resistance to ceftazidime (93.3%) and gentamicin (77.0%) (Table 5).

### 3.6. Associated Risk Factors Related to Neonates with Resistance to First Line Antibiotics

A regression analysis was completed looking for associations between neonatal sepsis, demographic, perinatal, clinical characteristics, and resistance to first-line antibiotic(s) (Table S4). Neonates admitted to YCH had a higher proportion of antibiotic resistance compared with CWH (79.2% versus 65.3%, respectively, *p* < 0.001). The proportion of antibiotic resistance increased over time from 2017 to 2019 (66.1% versus 81.1%, respectively, *p* = 0.005). Lastly, the proportion of first-line antibiotic resistance for Gram-negative pathogens was significantly greater than for Gram-positive (79.6% versus 64.1%, respectively, *p* < 0.001) (Table S4).

### 3.7. Multidrug-Resistance Pattern of Bacterial Isolates from Neonatal Septicemia

Most isolated pathogens also showed a high degree of resistance to reserve antibiotics such as extended-spectrum cephalosporins and carbapenems (Table 4 and Table 5) as well. Among those with bacteriologically confirmed sepsis, 393 (58.4%) were resistance to three or more different classes of antibiotics and considered to be MDR. Among Gram-positive isolates, 49% (123/251) were MDR, whereas 65% (272/421) of Gram-negative isolates were MDR (Table 6).

Results of drug resistance patterns compared within species specific Gram-positive isolates found that 30 (41.4%) of *S. aureus*, 24 (70.6%) of *S. epidermidis*, 62 (54.0%) of CoNS (others) and 6 (46.2%) of *Enterococcus* spp., were MDR isolates. A high proportion of *Burkholderia cepacia* (26/33, 78.8%), *Citrobacter* spp. (13/16, 81.2%), *K. pneumonia* (72/90, 80.0%), and *E. coli* (24/29, 82.8%) were MDR among Gram-negative isolates. There was no MDR patterns identified in the *Streptococcus* spp. The neonates with MDR infections were found to have a higher rate of unfavorable outcomes (died, left against medical advice, and discharged on request) than neonates with non-MDR infections (26% versus 16%) (Figure 1). Lastly, the case fatality rate of culture-positive sepsis caused by MDR isolates was higher than that of culture-positive sepsis caused by non-MDR isolates (65% versus 35%) (Table 6). 

### 3.8. Associated Risk Factors Related to Neonates with Unfavourable Treatment Outcomes among Neonates with Bacteriologically Confirmed Sepsis

Neonates admitted to CWH had a higher proportion of unfavorable outcomes compared to neonates admitted to YCH (29.3% versus 18.4%, respectively) (Table S5). Finally, we completed a regression analysis looking for associated risk factors (demographic, perinatal, and clinical characteristics) with unfavourable treatment outcomes. However, there were no significant associations revealed (Table S5).

## 4. Discussion

This is the first study in Myanmar looking at the frequency of neonatal bacterial sepsis, associated risk factors, degree of antibiotic resistance, and clinical outcomes among infants admitted to a NICU in two tertiary care facilities. The key findings from our study include: (1) 42% of neonates admitted for concern of sepsis were bacteriologically confirmed, (2) 74% of neonates with bacterial sepsis were resistant to first-line antibiotics, (3) the proportion of first-line antibiotic resistance progressed from 66% in the first year to 81% in the third year, (4) there was a higher proportion of neonates with antibiotic-resistant bacterial sepsis secondary to Gram-negative organisms (68%) than Gram-positive (37%), and (5) LOS was more frequent (45%) than EOS (38%). 

This study describes the frequency of neonatal bacterial sepsis in two large tertiary care NICUs, which is one of the principal causes of hospitalization and mortality for neonates in developing countries [22,23]. We found the prevalence of bacteriologically confirmed neonatal sepsis (672/10,935, 6.1%) in the two study facilities to be lower than reports from Bangladesh (14.5%) [24] and Nepal (14%) [25], but similar to one from India (6.2%) [26].

The proportion of neonatal EOS versus LOS was nearly equal between the two facilities (Table 2), although previous hospital data showed the prevalence of late onset sepsis was predominant in CWH [16]. There may be a disproportionate amount of EOS cases reported because neonates who presented with an episode of early onset sepsis and then later developed findings consistent with LOS during a prolonged hospital stay, were only counted as EOS. 

There were 42% of neonates with suspected bacteriologic sepsis confirmed by culture results. The frequency of culture-confirmed sepsis varies from 7% to 55%, depending upon geographical region [27,28,29]. Our blood culture-positivity rate was similar to previous local studies and other developing African and Asian countries [14,15,24,25,26,27,28,29]. The CDST results from blood cultures were not reported in 5.3% of our neonates (Figure 1). This may be due to several reasons including lack of adequate blood volume during sampling, processing challenges within the laboratories, or incorrect study ordering by providers.

The proportion of neonates found to be septic was significantly higher in the year 2017 and 2018 (compared to 2019). These findings might be related to the introduction of improved hand hygiene protocols in late 2018. Neonates delivered through emergency CS were more likely to have bacteriologically confirmed sepsis and this finding is consistent with other studies [30,31]. The possible reason could be related to increased risk of neonatal infection due to the surgical procedures, inadequate time for prophylactic antibiotics, prolonged hospital stays or health facility related infection control circumstances.

Among neonatal factors, very low and low birth weights (54.4%) were found to be associated with overall clinically suspected sepsis in this study (Table 2). Preterm birth and low birth weight were reported as associated risk factors of neonatal sepsis in previous studies as well [22,23,31,32]. However, others have found no association between neonatal sepsis and preterm birth or low birth weight [24,33]. Our findings suggest that those neonates with low or very low birth weight in this setting should be considered at higher risk for sepsis. 

There was no significant association between demographic, perinatal, and clinical characteristics with unfavorable treatment outcomes among neonates with bacteriologically confirmed sepsis (Table S5). In contrast, other studies have reported that preterm, low birth weight, late onset sepsis, home delivery, and Gram-negative sepsis were associated with unfavorable treatment outcomes [23,24,33]. These discordant findings may be due to the mixing of clinical factors from two different tertiary hospitals and other contextual variability. 

Among those neonates in this study who were blood culture-negative and suspected of having sepsis, the mortality rate was similar to those with culture-proven sepsis (16% vs 17% respectively). This may suggest that our culture-positivity rate should have been higher, as reported in other studies [27,28]. This could be related to intrapartum use of antibiotics, inadequate blood sampling, or sepsis due to other causes, such as viral or anaerobic organisms. Unfortunately, identifying these other types of sepsis remains challenging in this setting and further work needs to be done in broadening laboratory capabilities.

Our study found that septic neonates more frequently had a Gram-negative source of infection (68% versus 37%, respectively) and were typically late-onset clinically, similar findings from developing countries have been reported [23,29,34]. These two findings suggest that infections are more frequently not vertically related from the mother, but rather horizontally transmitted from individuals responsible for the care of the baby, the hospital environment, or community-acquired [27,28]. It may also indicate that at least some of these infections are hygiene-related, including poor infection control practices, which can be challenging in limited resource settings [35]. Thus, a key area of focus in trying to reduce the prevalence of neonatal sepsis could be improving infection control policies, including overall hygiene around routine and high-risk newborn care. 

In our study, most of the isolated bacteria were MDR (Table 6). Both Gram-negative and Gram-positive organisms were significantly resistant to commonly used antibiotic categories. Similar findings have been reported in previous studies [30,34,36]. The most frequent MDR organisms included *K. pneumoniae*, *B. cepacia*, and *A. baumannii*. Comparable findings were reported by others [12,27,30,37,38]. MDR in common pathogens raises the possibility of cross-transmission of mobile genetic elements that are able to jump across genera, including commensals [39]. *Acinetobacter* spp, CoNS, and *Klebsiella* spp., usually recognized as nosocomial pathogens, were the dominant pathogens in neonates with sepsis in this study. The high detection rate of uncommon nosocomial pathogens especially *Serratia* spp. and *Burkholderia* spp, was also reported by others [26,27,30,37].

In this study, the case fatality rate was higher (65%) among neonates with culture-positive sepsis secondary to MDR organisms compared with those caused by non-MDR organisms (35%, Table 6). These findings are similar to a study comparing neonatal mortality outcomes among those with MDR versus non-MDR sepsis (60% vs. 13%) [27].

Surprisingly, we found the mortality rate among culture-positive sepsis of approximately 17%, regardless of whether the neonate had bacteria resistant to a first-line antibiotic or not. A similar mortality rate for neonatal sepsis of 15% was recently reported in Uganda [36]. This would suggest that clinicians are very much aware of the high resistance rate in their NICU setting and are frequently choosing second-line antibiotics early in the course of management of neonatal sepsis. This frequency of first-line antibiotic resistance is similar to what has been reported by others [30,33]. These findings add evidence to the gap in knowledge regarding the prevalence of antibiotic resistance among neonates in the study setting.

Despite a decreasing frequency of NICU admissions for sepsis over the three years in the two facilities combined, 295 in 2017 versus 132 in 2019, the proportion of those neonates with first-line antibiotic resistance increased from 66% to 81% (*p* < 0.001). These results suggest an increasing burden of antibiotic resistance over time. These results are alarming as they predict possibly progressive increased resistance and need urgent further study to confirm this trend. 

The primary limitation of this study is that it only has three years of data from two NICUs within Yangon. It would be significantly more comprehensive if we could have included data from other NICUs around the country and have an extended timeline. Another concern is the possible low rate of culture-confirmed sepsis cases in comparison to other reports, which might suggest under-reporting or false-negative results. As noted previously, this may be related to technological limitations of the laboratories, sampling, and/or processing challenges. Specific antibiotic resistance patterns for each organism might be over or underestimated, because results are based on available data within each clinical setting. There also may be some degree of over classification of EOS or LOS, because of clinical interpretation of timing onset. Lastly, it is possible that the findings have some selection bias, as the two NICUs represent relatively high levels of care within Myanmar and the degree of AMR is likely higher than in smaller, community NICU facilities.

The greatest strength of the study is that much of the data was extracted from a newly standardized, systematic database (SERO), which has information on all neonates admitted to the study NICUs since 2016. In addition, we had a relatively large sample size to work with, including over 10,000 NICU admissions during the three-year study period. Lastly, the study followed the STROBE guidelines for observational studies [40]. Further research is needed to confirm the findings from this study. 

## 5. Conclusions

From 2017 to 2019, we found an increasing burden of antibiotic resistance within two tertiary hospital NICUs of Yangon, Myanmar. Among neonates with culture-proven sepsis, they were more likely to be of late clinical onset and secondary to Gram-negative pathogens with significant first-line antibiotic resistance. We also found a high proportion of multidrug-resistant pathogens that led to worse clinical outcomes. These findings suggest that a key policy change may be to focus on improved hygiene and infection control practices within these two hospitals and possibly other similar contexts. Emphasis must be placed on strategies for improved antimicrobial choices and stewardship within NICUs, in order to achieve reduction in infant mortality rate due to neonatal sepsis in Myanmar.

## Figures and Tables

**Table 1:** Operational definitions of types of sepsis and other operational definitions utilized in Yangon, Myanmar, January 2017–December 2019.

Neonatal Sepsis	Neonatal Sepsis Refers to Systemic and Generalized Bacterial Infection of the Newborn, Documented by a Positive Blood Culture in the First Four Weeks of Life.
Clinically suspected neonatal sepsis	Neonates with clinically suspected sepsis have a constellation of signs and symptoms that can include: fever, poor feeding, respiratory distress, cyanosis, tachycardia, seizures, hyperreflexia, jaundice, and temperature or blood pressure instability. All neonates in whom sepsis was suspected at CWH and YCH during the study period were included in the study.
Confirmed neonatal sepsis	Confirmed neonatal sepsis was identified as the presence of a clinically significant pathogen isolated from blood cultures with associated signs and symptoms.
Onset of sepsis	Neonatal sepsis can be categorized as early onset sepsis (EOS), with symptoms developing during the first 72 hours of life, or late onset sepsis (LOS) with symptoms starting after 72 hours of age.
Multidrug-resistant (MDR) organisms	Non-susceptibility to at least one agent in three or more antimicrobial categories was defined as multidrug-resistance (MDR) to groups such as penicillins, aminoglycosides, cephalosporins, fluoroquinolones, and carbapenems.

**Table 2:** Demographic, perinatal, and clinical characteristics of neonates with clinically suspected sepsis admitted to the NICU of two tertiary care hospitals in Yangon, Myanmar, January 2017 to December 2019, N = 1705.

Characteristics	YCH		CWH		Total		*p* Value $
	n	(%) *	n	(%) *	n	(%) *	
Total	1023	(100)	682	(100)	1705	(100)	
**Admission age in days**							
≤3	580	(56.7)	655	(96.0)	1235	(72.4)	<0.001
4–7	164	(16.0)	19	(2.8)	183	(10.7)	
8–28	279	(27.3)	8	(1.2)	287	(16.8)	
**Gender**							
Male	616	(60.2)	392	(57.5)	1008	(59.1)	0.260
Female	407	(39.8)	290	(42.5)	697	(40.9)	
**Year of admission**							
2017	434	(42.4)	298	(43.7)	732	(42.9)	0.020
2018	303	(29.6)	232	(34.0)	535	(31.4)	
2019	286	(28.0)	152	(22.3)	438	(25.7)	
**Gestational age**							
Preterm (<37 wk )	353	(34.5)	470	(68.9)	823	(48.3)	<0.001
Term (37 up to 42 wk )	642	(62.8)	210	(30.8)	852	(50.0)	
Post term (>42 wk )	9	(0.9)	2	(0.3)	11	(0.6)	
Missing	19	(1.9)	0	(0.0)	19	(1.1)	
**PROM (>18 h)**							
Yes	273	(26.7)	168	(24.6)	441	(25.9)	0.343
No	750	(73.3)	514	(75.4)	1264	(74.1)	
**Place of Delivery**							
Institutional	802	(78.4)	682	(100.0)	1484	(87.0)	<0.001
Home	221	(21.6)	0	(0.0)	221	(13.0)	
**Mode of delivery**							
Normal	576	(56.3)	285	(41.8)	861	(50.5)	<0.001
Forceps/Vacuum	15	(1.5)	22	(3.2)	37	(2.2)	
Elective Caesarean section	54	(5.3)	19	(2.8)	73	(4.3)	
Emergency Caesarean section	378	(37.0)	356	(52.2)	734	(43.0)	
**Delivery conducted by**							
Doctor	755	(73.8)	678	(99.4)	1433	(84.0)	<0.001
Others ^#^	268	(26.2)	4	(0.6)	272	(16.0)	
**Meconium staining liquor**							
Yes	75	(7.3)	150	(22.0)	225	(13.2)	<0.001
No	948	(92.7)	532	(78.0)	1480	(86.8)	
**Foul smelling liquor**							
Yes	70	(6.8)	24	(3.5)	94	(5.5)	0.003
No	953	(93.2)	658	(96.5)	1611	(94.5)	
**Birth Asphyxia**							
Yes	186	(18.2)	233	(34.2)	419	(24.6)	<0.001
No	763	(74.6)	449	(65.8)	1212	(71.1)	
Missing	74	(7.2)	0	(0.0)	74	(4.3)	
**Birth weight**							
Very low (<1500 g)	127	(12.4)	272	(39.9)	399	(23.4)	<0.001
Low (1500 g to 2500 g)	294	(28.7)	235	(34.5)	529	(31.0)	
Normal (>2500 g)	602	(58.8)	175	(25.7)	777	(45.6)	
**Hyperbilirubinemia (>20 mg per dL)**							
Yes	459	(44.9)	463	(67.9)	922	(54.1)	<0.001
No	564	(55.1)	219	(32.1)	783	(45.9)	
**Hypothermia (Temperature < 35.5 °C)**							
Yes	260	(25.4)	10	(1.5)	270	(15.8)	<0.001
No	763	(74.6)	672	(98.5)	1435	(84.2)	
**Onset**							
Early	503	(49.2)	333	(48.8)	836	(49.0)	0.889
Late	520	(50.8)	349	(51.2)	869	(51.0)	

YCH = Yangon Children Hospital; CWH= Central Women Hospital; NICU = Neonatal intensive care unit; PROM = Prolonged rupture of membrane; Birth Asphyxia = (For extramural/outborn babies, YCH): Slow gasping breathing at 1-minute of age, and (For intramural/inborn babies, CWH): Apgar score of less than 7 at 1 min of age; Other# = Nurse, Midwife, Traditional Birth Attendant, Skilled Birth Attendant, and Self; * Column percentage. $ Chi-square test.

**Table 3:** Turn-around time of CDST among neonates with clinically suspected sepsis admitted to the NICU of two tertiary care hospitals in Yangon, Myanmar, January 2017 to December 2019.

Time Variable	Count
**Turn-Around Time (n = 1533)**	**Median Days (IQR)**
Admission and CDST test	2 (2–4)
CDST test and receipt of results	6 (6–6)
Admission to CDST results	7 (6–9)
**Time to Reporting CDST Results (Days)**	**n (%)**
≤ 6	474 (29.3)
7–14	968 (60.0)
≥15	91 (5.6)
Missing	82 (5.1)

CDST = culture and drug susceptibility testing, NICU = neonatal intensive care unit, IQR = interquartile range.

**Table 4:** Profile of Gram-positive isolates and antimicrobial resistance pattern among neonates with bacteriologically confirmed sepsis admitted to the NICU of two tertiary care hospitals in Yangon, Myanmar, January 2017 to December 2019.

Drugs	*S. aureus*		*S. epidermidis*		CoNS (Others)	*Enterococcus* spp.			Others *		Total	
	n/N	(%)	n/N	(%)	n/N	(%)	n/N	(%)	n/N	(%)	n/N	(%)
**Total**	**73**		**34**		**115**		**13**		**16**		**251**	
Ampicillin	37/43	(86.0)	21/23	(91.3)	43/49	(87.8)	4/10	(40.0)	4/14	(28.6)	109/139	(78.4)
Cloxacillin	12/20	(60.0)	18/22	(81.8)	35/47	(74.5)	0/1	(0.0)	1/1	(100.0)	66/101	(65.3)
Amoxi-Clav	11/27	(40.7)	24/29	(82.8)	47/62	(75.8)	4/6	(66.7)	NT	NT	86/134	(64.2)
Gentamicin	21/67	(31.3)	12/28	(42.9)	28/111	(25.2)	3/6	(50.0)	3/7	(17.6)	67/229	(29.3)
Amikacin	2/30	(6.7)	2/5	(40.0)	0/28	(0.0)	3/6	(50.0)	1/1	(100.0)	8/70	(11.4)
Cefotaxime	15/44	(34.0)	7/21	(33.3)	40/81	(49.4)	4/5	(80.0)	0/3	NT	66/154	(42.9)
Ceftazidime	3/12	(25.0)	19/24	(79.2)	14/21	(66.7)	2/2	(100.0)	NT	NT	38/59	(64.4)
Cefoperazone#	3/7	(42.9)	14/20	(70.0)	9/25	(60.0)	2/2	(100.0)	0/1	(0.0)	28/45	(62.2)
Vancomycin	4/27	(14.8)	2/23	(8.7)	13/64	(20.3)	0/7	(0.0)	0/11	(0.0)	19/132	(14.4)
PT	10/34	(29.4)	21/27	(77.8)	17/38	44.7)	2/5	(40.0)	NT	NT	50/104	(48.1)
Imipenem	7/34	(20.6)	19/25	(76.0)	18/38	(47.4)	0/2	(0.0)	1/4	(25.0)	45/103	(43.7)
Meropenem	3/15	(20.0)	17/21	(81.0)	13/21	(61.9)	1/2	(50.0)	0/2	(0.0)	34/61	(55.7)
Linezolid	3/43	(7.0)	0/23	(0.0)	5/75	(6.7)	0/6	(0.0)	1/8	(12.5)	9/155	(5.8)
Ciprofloxacin	13/47	(27.7)	7/25	(28.0)	31/62	(50.0)	4/4	(100.0)	2/8	(25.0)	57/146	(39.0)
Levofloxacin	6/36	(16.7)	8/24	(33.3)	30/76	(39.5)	5/10	(50.0)	5/11	(45.5)	54/157	(34.4)
Erythromycin	15/25	(60.0)	12/21	(57.1)	30/49	(61.2)	4/7	(57.1)	2/8	(25.0)	63/110	(57.3)
Co-trimoxazole	7/21	(33.3)	7/17	(41.2)	16/41	(39.0)	4/15	(80.0)	0/4	(0.0)	34/88	(38.6)

Data are n/N (%); there are variations in denominators in few cells as antibiotic sensitivity testing for all drugs was not done. N = number of isolates with resistance; N = Number of isolates tested for DST; PT = Piperacillin-Tazobactam; Amoxi-Clav = Amoxicillin clavulanic acid; NT = Not tested. NICU = Neonatal intensive care unit; *S. aureus* = *Staphylococcus aureus*; *S. epidermidis* = *Staphylococcus epidermidis*; CoNS= Coagulase Negative *Staphylococcus* excluding *S. epidermidis*; #= Cefoperazone or Cefoperazone-sulbactam.* Others = *Streptococcus* species, *Bacillus* species, *Corynebacterium kutscheri*, *Corynebacterium* spp, *Streptococcus mutans*, *Kocuria rosea*, *Micrococcus luteus*, *Aerococcus nishinomiyaensis*, *Clostridium subterminalae*, *Streptococcus gallolyticus*, *Streptococcus mitis*, *Streptococcus pyogenes*.

**Table 5:** Gram-negative isolates and antimicrobial resistance pattern among neonates with bacteriologically confirmed sepsis admitted to the NICU of two tertiary care hospitals in Yangon, Myanmar, January 2017 to December 2019.

Drugs	*K. pneumoniae*		*S. marcescens*		*B. cepacia*		*P. aeruginosa*		*Acinetobacter* spp.		*Enterobacter* spp.		Others *		Total	
	n/N	(%)	n/N	(%)	n/N	(%)	n/N	(%)	n/N	(%)	n/N	(%)	n/N	(%)	n/N	(%)
**Total**	**90**		**66**		**33**		**33**		**40**		**36**		**123**		**421**	
Ampicillin	59/63	(93.7)	30/30	(100.0)	20/20	(100.0)	5/5	(100.0)	19/19	(100.0)	24/24	(100.0)	60/67	(89.6)	217/228	(95.2)
Cloxacillin	0/1	(0.0)	1/2	(50.0)	0/1	(0.0)	0/5	(0.0)	0/1	(0.0)	0/1	(0.0)	0/2	(0.0)	1/2	(50.0)
Amoxi-Clav	27/45	(60.0)	49/50	(98.0)	10/12	(83.3)	5/15	(33.0)	9/12	(75.0)	21/24	(87.5)	31/61	(50.8)	152/219	(69.4)
Gentamicin	57/74	(77.0)	22/31	(71.0)	21/25	(84.0)	17/18	(94.0)	11/37	(30.0)	15/25	(60.0)	53/104	(51.0)	196/314	(62.4)
Amikacin	10/82	(12.2)	29/59	(49.0)	22/30	(73.3)	14/28	(50.0)	6/25	(24.0)	8/28	(28.6)	28/107	(26.2)	117/359	(32.6)
Cefotaxime	40/73	(54.8)	30/55	(55.0)	16/23	(69.6)	5/21	(24.0)	6/23	(26.0)	16/27	(59.2)	27/81	(33.3)	140/303	(46.2)
Ceftazidime	56/60	(93.3)	17/32	(53.0)	9/19	(47.4)	5/12	(42.0)	9/16	(56.0)	10/18	(55.5)	42/76	(55.3)	148/233	(63.5)
Cefoperazone#	13/60	(21.7)	7/11	(64.0)	2/15	(13.3)	1/2	(50.0)	4/8	(50.0)	2/6	(33.3)	5/13	(38.5)	34/115	(29.6)
PT	11/68	(16.2)	21/33	(64.0)	12/27	(44.4)	1/17	(5.9)	7/31	(23.0)	11/27	(40.7)	24/85	(28.2)	87/288	(30.2)
Imipenem	4/67	(6.0)	10/23	(44.0)	20/23	(87.0)	12/26	(46.0)	4/27	(15.0)	5/23	(21.7)	26/88	(29.5)	81/277	(29.2)
Meropenem	9/73	(12.3)	17/53	(32.0)	5/25	(20.0)	0/8	(0.0)	2/22	(9.1)	7/24	(29.2)	20/72	(27.8)	60/277	(21.7)
Ciprofloxacin	17/62	(27.4)	4/43	(9.3)	15/27	(55.6)	2/27	(7.4)	1/27	(3.7)	0/23	(0.0)	21/87	(24.2)	60/296	(20.3)
Levofloxacin	11/73	(15.1)	2/57	(3.5)	5/32	(15.6)	0/12	(0.0)	2/24	(5.9)	1/31	(3.2)	14/101	(13.9)	35/340	(10.3)
Co-trimoxazole	34/63	(64.2)	4/45	(8.9)	1/18	(5.6)	2/3	(67.0)	4/5	(80.0)	6/18	(33.3)	23/66	(34.8)	74/218	(33.9)

PT = Piperacillin-Tazobactam; Amoxi-Clav = Amoxicillin clavulanic acid; n = number of isolates with resistance; N = Number of isolates tested for DST. # = Cefoperazone or Cefoperazone-Sulbactam. *K. pneumonia* = *Klebsiella pneumonia*; *S. marcescens* = *Serratia marcescens*; *P. aeruginosa* = *Pseudomonas aeruginosa*; *B. cepacia* = *Burkholderia cepacia*. * Others = *Klebsiella* species, *Coliform* species, *Escherichia coli*, *Raoultella ornithinolytica*, *Pseudomonas oleovorans*, *Pseudomonas* species, *Proteus mirabilis*, *Serratia liquefaciens*, *Citrobacter* species, *Kluyvera intermedia*, *Serratia* species, *Pantoea* spp, *Elizabethkingia meningoseptica*, *Aeromonas hydrophila*, *Kluyvera cryocrescens*, *Sphingomonas paucimobilis*, *Aerococcus nishinomiyaensis*, *Brevundimonas diminuta*, *Pantoea agglomerans*, *Klebsiella ozaenae*, *Serratia phymuthica*, *Serratia rubidaea*, *Aeromonas veronii* biovar sobia, *Stenotrophomonas maltophilia*, *Roseomonas gilardii*, *Pasteurella pneumotrobia*, *Pluralibacter gergoviae*.

**Table 6:** Multidrug-resistance and case-fatality rate (CFR) of bacterial isolates from neonatal septicemia admitted to the NICU of two tertiary care hospitals in Yangon, Myanmar, January 2017 to December 2019.

Neonatal Bloodstream Pathogens	No. of Isolates	MDR Isolates (Number, %)	Non-MDR Isolates (Number, %)	CFR Due to MDR in Culture-Positive Isolates (n/N, %)	CFR Due to Non-MDR in Culture-Positive Isolates (n/N, %)
**Gram-positive organisms**	251	123 (49.0)	128 (51.0)	19/28 (67.9)	9/28 (32.1)
*S. aureus*	73	30 (41.1)	43 (59.0)	6/10 (60.0)	4/10 (40.0)
*S. epidermidis*	34	24 (70.6)	10 (29.4)	2/2 (100)	0/0 (0.0)
CoNS (others)	115	62 (54.0)	53 (46.1)	10/14 (71.4)	4/14 (28.6)
*Enterococcus* spp.	13	6 (46.2)	7 (53.8)		
*Streptococcus* spp.	8	0	8 (100)		
Others Gram-positive organisms	8	1 (12.5)	7 (87.5)	1/1 (100)	0/7 (0.0)
**Gram-negative organisms**	421	272 (64.6)	149 (35.4)	54/84 (64.3)	30/84 (35.7)
*Klebsiella pneumoniae*	90	72 (80.0)	18 (20.0)	16/20 (80.0)	4/20 (20.0)
*Serratia marcescens*	66	42 (63.7)	24 (36.4)	4/8 (50.0)	4/8 (50.0)
Other *Serratia* spp.	16	8 (50.0)	8 (50.0)	4/5 (80.0)	1/5 (20.0)
*Burkholderia cepacia*	33	26 (78.8)	7 (21.2)		
*Pseudomonas aeruginosa*	33	16 (48.5)	17 (51.5)	5/9 (55.5)	4/9 (44.5)
*Acinetobacter baumannii*	29	13 (44.8)	16 (55.2)	5/7 (71.4)	2/7 (28.6)
*Enterobacter* spp.	36	26 (69.4)	10 (30.5)	5/9 (55.5)	4/9 (44.5)
*Citrobacter* spp.	16	13 (81.2)	3 (18.8)	5/5 (100)	0/5 (0.0)
Other Gram-negative bacilli	44	17 (40.9)	27 (59.1)	3/9 (33.3)	6/9 (66.6)
*E.coli*	29	24 (82.8)	5 (17.2)	7/8 (87.5)	1/8 (12.5)
Other *Acinobacter* spp.	11	4 (36.4)	7 (63.6)	0/3 (0.0)	3/3 (100)
Other *Klebsiella* spp.	18	11 (61.1)	7 (38.9)	0/1 (0.0)	1/1 (100)
Total	672	395 (58.8)	277 (41.2)	73/112 (65.2)	39/112 (34.8)

n/N; n = number of deaths due to MDR /non-MDR for each group of organism, N = number of total dead due to MDR /non-MDR for each group of organism.

## Supplementary Materials

The following are available online at https://www.mdpi.com/article/10.3390/tropicalmed6020062/s1, Table S1: Distribution of all bacteria isolates from positive blood culture (n = 672), Table S2: Bacteriological profile found in positive blood culture (n = 672) from neonate with early and late onset sepsis, Table S3: Demographic, perinatal and clinical characteristics associated with culture positive bacteriological sepsis among neonates with clinically suspected sepsis admitted to the NICU of two tertiary care hospitals in Yangon, Myanmar from January 2017 to December 2019, N = 1615, Table S4: Demographic, perinatal and clinical characteristics associated with resistance to at least one first-line antibiotic among neonates with confirmed sepsis admitted to the NICU of two tertiary care hospitals in Yangon, Myanmar, January 2017 to December 2019, Table S5: Demographic, perinatal and clinical characteristics associated with unfavourable treatment outcomes among neonates with bacteriologically confirmed sepsis admitted to the NICU of two tertiary care hospitals in Yangon, Myanmar from January 2017 to December 2019, N = 672.

## Open Access Statement

In accordance with WHO’s open-access publication policy for all work funded by WHO or authored/co-authored by WHO staff members, the WHO retains the copyright of this publication through a Creative Commons Attribution IGO license (http://creativecommons.org/licenses/by/4.0/igo/legalcode, accessed on 18 April 2021) which permits unrestricted use, distribution and reproduction in any medium provided the original work is properly cited.
