# Supplementary material for: Neonatal Sepsis, Antibiotic Susceptibility Pattern, and Treatment Outcomes among Neonates Treated in Two Tertiary Care Hospitals of Yangon, Myanmar from 2017 to 2019
Source: Trop Med Infect Dis. 2021 Apr 28;6(2):62. doi: 10.3390/tropicalmed6020062 (PMC8167801; doi:10.3390/tropicalmed6020062)
Supplement: Supplementary file 1 [file tropicalmed-06-00062-s001.zip › tropicalmed-1159848-supplementary.pdf]

Tropical Medicine and Infectious Disease

MDPI

Supplementary Materials of Neonatal Sepsis, Antibiotic Susceptibility Pattern and Treatment Outcomes among Neonates Treated in Two Tertiary Care Hospitals of Yangon, Myanmar from 2017 to 2019

Table 1. Distribution of all bacteria isolates from positive blood culture (n= 672).

Isolated bacteria	Total	(%)
CoNS (others)	115	(17.1)
Klensiella pneumoniae	90	(13.4)
S. aureus	73	(10.9)
Serratia marcescens	66	(9.8)
Other gram negative bacilli	11	(1.6)
Enterobacter spp.	36	(5.4)
S. epidermidis(CoNS)	34	(5.1)
Burkholderia cepacia	33	(4.9)
Pseudomonas aeruginosa	33	(4.9)
Acinetobacter baumannii	29	(4.3)
E.coli	29	(4.3)
Klebsiella spp.	18	(2.7)
Serratia spp.	16	(2.4)
Citrobacter spp.	16	(2.4)
Enterococcus spp.	13	(1.9)
Acinobacter spp.	11	(1.6)
Streptococcus spp.	8	(1.2)
Others Gram positive	8	(1.2)
Pantoea spp.	5	(0.7)
Coliform spp.	6	(0.9)
Aeromonas spp.	4	(0.6)
Proteus mirabilis	4	(0.6)
Pseudomonas spp.	5	(0.7)
Kluyvera cryocrescens	4	(0.6)
Elizabethkingia meningoseptica	3	(0.4)
Stenotrophomonas maltophilia	2	(0.3)
Total	672	(100)

Table 2. Bacteriological profile found in positive blood culture (n = 672) from neonate with early and late onset sepsis.

Isolated bacteria	EOS, (number, %)	LOS, (number, %)	Total
**Gram-positive isolates**	**117 (46.6)**	**134 (53.4)**	**251**
S. aureus	37 (50.7)	36 (49.3)	73
S epidermidis	10 (29.4)	24 (70.6)	34
CoNS (others)	56 (48.7)	59 (51.3)	115
Enterococcus spp.	7 (53.8)	6 (46.2)	13
Streptococcus spp.	3 (50.7)	5 (62.5)	8
Others gram-positive isolates	4 (50.0)	4 (50.0)	8
**Gram-negative isolates**	**177 (42.0)**	**244 (58.0)**	**421**

Klebsiella pneumoniae	35	(38.9)	55	(61.1)	90
Serratia mercencus	28	(50.7)	38	(57.6)	66
Other *Serratia* spp.	10	(62.5)	6	(37.5)	16
Burkholderia cepacia	12	(36.4)	21	(63.6)	33
Pseudomonas aeruginosa	19	(57.6)	14	(42.4)	33
Acinetobacter baumannii	5	(50.7)	24	(50.7)	29
Enterobacter spp.	18	(50.0)	18	(50.0)	36
Citrobacter spp.	11	(68.8)	5	(31.3)	16
Other gram-negative bacilli	15	(43.1)	29	(65.9)	44
E.coli	10	(34.5)	19	(65.5)	29
Other *Acinetobacter* spp.	4	(36.4)	7	(63.6)	11
Other *Klebsiella* spp.	10	(55.6)	8	(44.4)	18
**Total**	**294 (43.7)**		**378 (56.3)**		**672**

Table 3. Demographic, perinatal and clinical characteristics associated with culture positive bacteriological sepsis among neonates with clinically suspected sepsis admitted to the NICU of two tertiary care hospitals in Yangon, Myanmar from January 2017 to December 2019, N= 1615.

Characteristics	Total	Culture positive		Unadjusted		Adjusted#		p Value
		n	(%)*	PR	(95% CI)	PR	(95% CI)	
Total	1615	672	(41.6)					
**Age in days**								
≤ 3	1160	492	(42.4)	1.0		1.0		
4-7	179	77	(43.0)	1.0	(0.8-1.2)	1.0	(0.8-1.2)	0.975
8-28	276	103	(37.3)	0.9	(0.8-1.0)	0.9	(0.7-1.1)	0.220
**Gender**								
Male	946	392	(41.4)	1.0				
Female	669	280	(41.9)	1.0	(0.9-1.1)			
**Hospital admitted**								
YCH	990	413	(41.7)	1.0	(0.9-1.1)			
CWH	625	259	(41.4)	1.0				
**Year of admission**								
2017	685	295	(43.1)	1.4	(1.2-1.7)	1.4	(1.2-1.7)	<0.001
2018	497	245	(49.3)	1.6	(1.4-1.9)	1.5	(1.3-1.8)	<0.001
2019	433	132	(30.5)	1.0		1.0		
**Gestational age**								
Preterm (<37 wk)	789	392	(49.7)	1.5	(1.3-1.7)	1.2	(1.0-1.5)	0.094
Term (37 up to 42 wk)	797	267	(33.5)	1.0		1.0		
Post term (>42 wk)	10	5	(50.0)	1.5	(0.8-2.7)	1.5	(0.9-2.7)	0.128
Missing	19	8	(42.1)	1.3	(0.7-2.1)	1.1	(0.6-1.9)	0.818
**PROM (>18hr)**								
Yes	429	196	(45.7)	1.1	(1.0-1.3)	1.0	(0.9-1.1)	0.978
No	1186	476	(40.1)	1.0		1.0		
**Place of Delivery**								

Institutional	1398	584	(41.8)	1.0	(0.9-1.2)			
Home	217	88	(40.6)	1.0				
**Mode of delivery**								
Normal	821	313	(38.1)	1.0		1.0		
Forceps/Vaccum	32	10	(31.3)	0.8	(0.5-1.4)	1.1	(0.6-1.9)	0.777
Elective CS	70	28	(40.0)	1.0	(0.8-1.4)	1.1	(0.8-1.5)	0.434
Emergency CS	692	321	(46.4)	1.2	(1.1-1.4)	1.2	(1.1-1.4)	0.001
**Delivery conducted by**								
Doctor	1347	567	(42.1)	1.1	(0.9-1.3)			
Others#	268	105	(39.2)	1.0				
**Meconium staining liquor**								
Yes	204	61	(29.9)	1.0		1.0		
No	1411	611	(43.3)	1.4	(1.2-1.8)	1.2	(0.9-1.5)	0.166
**Foul smelling liquor**								
Yes	86	38	(44.2)	1.1	(0.8-1.4)			
No	1529	634	(41.5)	1.0				
**Birth Asphyxia**								
Yes	393	140	(35.6)	1.0		1.0		
No	1150	500	(43.5)	1.2	(1.1-1.4)	1.2	(1.0-1.4)	0.010
Missing	72	32	(44.4)	1.2	(0.9-1.7)	1.3	(1.0-1.7)	0.062
**Birth weight**								
Very low (<1.5kg)	379	205	(54.1)	1.6	(1.4-1.8)	1.2	(1.0-1.6)	0.073
Low (1.5 to 2.5kg)	512	223	(43.6)	1.3	(1.1-1.5)	1.1	(0.9-1.3)	0.544
Normal (>2.5kg)	724	244	(33.7)	1.0		1.0		
**Hyperbilirubinemia (> 20 mg per dL)**								
Yes	870	368	(42.3)	1.0	(0.9-1.2)			
No	745	304	(40.8)	1.0				
**Hypothermia (<37.5 °C)**								
Yes	266	120	(45.1)	1.1	(1.0-1.3)	1.1	(1.0-1.3)	0.141
No	1349	552	(40.9)	1.0		1.0		
**Onset**								
Early	779	294	(37.7)	1.0		1.0		
Late	836	378	(45.2)	1.2	(1.1-1.3)	1.2	(1.1-1.4)	0.008

YCH= Yangon Children Hospital; CWH= Central Women Hospital; NICU= Neonatal intensive care unit; PR- Prevalence ratio; CI- Confidence interval; PROM= Prolonged rupture of membrane; Birth Asphyxia= (For extramural/outborn babies, YCH): Slow gasping breathing at 1-minute of age and (For intramural/inborn babies, CWH): Apgar score of less than 7 at 1 minute of age; Other#= Nurse, Midwife, Traditional Birth Attendant, Skilled Birth Attendent and Self; * Row percentage; # Modified Poisson regression.

Table 4. Demographic, perinatal and clinical characteristics associated with resistance to at least one first-line antibiotic among neonates with confirmed sepsis admitted to the NICU of two tertiary care hospitals in Yangon, Myanmar, January 2017 to December 2019.

Characteristics	Total	Resistance*		Unadjusted		Adjusted $		p Value
		n	(%)*	PR	(95% CI)	PR	(95% CI)	

**Total**	**672**	**496**	**(73.8)**					
**Admission age in days**								
≤ 3	492	365	(74.2)	1.0		1.0		
4-7	77	55	(71.4)	1.0	(0.8-1.1)	1.0	(0.8-1.1)	0.521
8-28	103	76	(73.8)	1.0	(0.9-1.1)	1.0	(0.8-1.1)	0.506
**Gender**								
Male	392	285	(72.7)	1.0				
Female	280	211	(75.4)	1.0	(0.9-1.1)			
**Hospital admitted**								
YCH	413	327	(79.2)	1.2	(1.1-1.3)	1.2	(1.1-1.4)	<0.001
CWH	259	169	(65.3)	1.0		1.0		
**Year of admission**								
2017	295	195	(66.1)	1.0		1.0		
2018	245	194	(79.2)	1.2	(1.1-1.3)	1.1	(1.0-1.3)	0.019
2019	132	107	(81.1)	1.2	(1.1-1.4)	1.2	(1.1-1.3)	0.005
**Gestational age**								
Preterm (<37 wk)	392	293	(74.7)	1.0	(0.9-1.1)			
Term (37 up to 42 wk)	267	192	(71.9)	1.0				
Post term (>42 wk)	5	3	(60)	0.8	(0.4-1.7)			
Missing	8	8	(100)	-				
**PROM (>18hr)**								
Yes	196	147	(75.0)	1.0	(0.9-1.1)			
No	476	349	(73.3)	1.0				
**Place of Delivery**								
Institutional	584	427	(73.1)	1.0		1.0		
Home	88	69	(78.4)	1.1	(1.0-1.2)	1.0	(0.9-1.1)	0.870
**Mode of delivery**								
Normal	313	236	(75.4)	1.0				
Forceps/Vacuum	10	8	(80.0)	1.1	(0.8-1.5)			
Elective CS	28	21	(75.0)	1.0	(0.8-1.2)			
Emergency CS	321	231	(72.0)	1.0	(0.9-1.0)			
**Delivery conducted by**								
Doctor	567	400	(70.3)	1.0	(0.9-1.1)			
Others[#]	105	79	(71.8)	1.0				
**Meconium staining liquor**								
Yes	61	44	(72.1)	1.0				
No	611	452	(74.0)	1.0	(0.9-1.2)			
**Foul smelling liquor**								
Yes	38	28	(73.7)	1.0	(0.8-1.2)			
No	634	468	(73.8)	1.0				

**Birth Asphyxia**								
Yes	140	98	(70.0)	1.0		1.0		
No	500	371	(74.2)	1.1	(0.9-1.2)	1.0	(0.9-1.2)	0.682
Missing	32	27	(84.4)	1.2	(1.0-1.4)	1.0	(0.8-1.2)	0.06
**Birth weight**								
Very low (<1.5kg)	205	156	(76.1)	1.1	(1.0-1.2)	1.1	(1.0-1.3)	0.152
Low (1.5 to 2.5kg)	223	170	(76.2)	1.1	(1.0-1.2)	1.1	(0.9-1.2)	0.133
Normal (>2.5kg)	244	170	(69.7)	1.0		1.0		
**Hyperbilirubinemia (> 20 mg per dL)**								
Yes	368	265	(72.0)	1.0		1.0		
No	304	231	(76.0)	1.1	(1.0-1.2)	1.1	(0.9-1.2)	0.103
**Hypothermia (Temperature <35.5 °C)**								
Yes	120	103	(85.8)	1.2	(1.1-1.3)	1.0	(0.9-1.2)	0.492
No	552	393	(71.2)	1.0		1.0		
**Onset**								
Early	294	217	(73.8)	1.0				
Late	378	279	(73.8)	1.0	(0.9-1.1)			
**Bacteriological isolate**								
Gram-positive	251	161	(64.1)	1.0		1.0		
Gram-negative	421	335	(79.6)	1.2	(1.1-1.4)	1.2	(1.1-1.3)	<0.001

CDST= Culture and drug susceptibility testing; NICU= Neonatal intensive care unit; PR= Prevalence ratio; Ci= Confidence interval; PROM= Prolonged rupture of membrane; Birth Asphyxia= (For extramural/outborn babies, YCH): Slow gasping breathing at 1-minute of age and (For intramural/inborn babies, CWH): Apgar score of less than 7 at 1 minute of age; Other#= Nurse, Midwife, Traditional Birth Attendant, Skilled Birth Attendent and Self;. * Resistance to at least one first line antibiotics- ampicillin, amikacin, gentamicin and cefotaxime; # Row percentage; $ Modified Poisson Regression.

Table 5. Demographic, perinatal and clinical characteristics associated with unfavourable treatment outcomes among neonates with bacteriologically confirmed sepsis admitted to the NICU of two tertiary care hospitals in Yangon, Myanmar from January 2017 to December 2019, N= 672.

Characteristics	Total	Unfavourable*		Unadjusted		Adjusted#		p Value
		n	(%)*	PR	(95% CI)	PR	(95% CI)	
Total	672	152	(22.6)					
**Admission age in days**								
≤ 3	492	121	(24.6)	1.4	(0.9-2.2)	1.1	(0.6-1.9)	0.762
4-7	77	13	(16.9)	1.0	(0.5-1.8)	1.0	(0.5-1.9)	0.960
8-28	103	18	(17.5)	1.0				
**Gender**								
Male	392	92	(23.5)	1.1	(0.8-1.5)			
Female	280	60	(21.4)	1.0				
**Hospital admitted**								
YCH	413	76	(18.4)	1.0		1.0		
CWH	259	76	(29.3)	1.6	(1.2-2.1)	1.1	(0.7-1.6)	0.709
**Year of admission**								
2017	295	66	(22.4)	1.3	(0.9-2.1)	1.4	(0.9-2.2)	0.095

2018	245	64	(26.1)	1.6	(1.0-2.4)	1.4	(0.9-2.2)	0.107
2019	132	22	(16.7)	1.0		1.0		
**Gestational age**								
Preterm (<37 wk)	392	113	(28.8)	2.1	(1.5-3.0)	1.5	(0.9-2.6)	0.164
Term (37 up to 42 wk)	267	36	(13.5)	1.0		1.0		
Post term (>42 wk)	5	0	(0.0)	-		-		
Missing	8	3	(37.5)	2.8	(1.1-7.2)	2.5	(0.9-6.8)	0.066
**PROM (>18hr)**								
Yes	196	51	(26.0)	1.2	(0.9-1.6)	1.0	(0.7-1.5)	0.827
No	476	101	(21.2)	1.0		1.0		
**Place of Delivery**								
Institutional	584	134	(22.9)	1.1	(0.7-1.7)			
Home	88	18	(20.5)	1.0				
**Mode of delivery**								
Normal	313	68	(21.7)	1.0		1.0		
Forceps/Vacuum	10	1	(10.0)	0.5	(0.1-3.0)	0.8	(0.1-5.2)	0.831
Elective CS	28	3	(10.7)	0.5	(0.2-1.5)	0.6	(0.2-1.7)	0.298
Emergency CS	321	80	(24.9)	1.1	(0.9-1.5)	1.2	(0.9-1.6)	0.236
**Delivery conducted**								
Doctor	567	130	(22.9)	1.1	(0.7-1.6)			
Others[#]	105	22	(21.0)	1.0				
**Meconium staining**								
Yes	61	8	(13.1)	1.0				
No	611	144	(23.6)	1.8	(0.9-3.5)	1.4	(0.7-2.8)	0.305
**Foul smelling**								
Yes	38	8	(21.1)	0.9	(0.5-1.7)			
No	634	144	(22.7)	1.0				
**Birth Asphyxia**								
Yes	140	35	(25.0)	1.1	(0.8-1.6)			
No	500	109	(21.8)	1.0				
Missing	32	8	(25.0)	1.1	(0.6-2.1)			
**Birth weight**								
Very low (<1.5kg)	205	68	(33.2)	2.4	(1.6-3.4)	1.5	(0.9-2.6)	0.130
Low (1.5kg to 2.5kg)	223	50	(22.4)	1.6	(1.1-2.4)	1.1	(0.7-1.9)	0.675
Normal (>2.5kg)	244	34	(13.9)	1.0		1.0		
**Hyperbilirubinemia (> 20 mg per dL)**								
Yes	368	78	(21.2)	0.9	(0.7-1.2)			
No	304	74	(24.3)	1.0				
**Hypothermia (Temperature <35.5 °C)**								
Yes	120	18	(15.0)	0.6	(0.4-1.0)	0.6	(0.3-0.9)	0.029
No	552	134	(24.3)	1.0		1.0		

**Bacteriological isolate**								
Gram-positive	251	43	(17.1)	1.0		1.0		
Gram- negative	421	109	(25.9)	1.5	(1.1-2.1)	1.1	(0.8-1.6)	0.505
**Onset**								
Early	294	60	(20.4)	1.0		1.0		
Late	378	92	(24.3)	1.2	(0.9-1.6)	1.1	(0.8-1.5)	0.46
**Resistance** $								
Yes	496	119	(24.0)	1.3	(0.9-1.8)	1.3	(1.0-1.9)	0.094
No	176	33	(18.8)	1.0		1.0		

CDST= Culture and drug susceptibility testing; NICU= Neonatal intensive care unit; PR= Prevalence ratio; CI= Confidence interval; PROM= Prolonged rupture of membrane; Birth Asphyxia= (For extramural/out born babies, YCH): Slow gasping breathing at 1-minute of age and (For intramural/inborn babies, CWH): Apgar score of less than 7 at 1 minute of age; Other#= Nurse, Midwife, Traditional Birth Attendant, Skilled Birth Attendant and Self;. * Unfavourable- Died, Left Against Medical Advice, Discharge on Request, Referred out; # Modified Poisson Regression; $ First line antibiotics- ampicillin, amikacin, gentamicin and cefotaxime.
